# The interaction of biological network topology and mutation effects in complex trait evolution

**DOI:** 10.3389/fsysb.2026.1845370

**Published:** 2026-07-10

**Authors:** Alexandre Gouy

**Affiliations:** AlgoLife SARL, Saint-Julien-le-Petit, France

**Keywords:** complex traits, gene regulatory networks, genotype-phenotype map, mutation effects, polygenic architecture

## Abstract

The genetic architecture of complex traits can be described through three complementary frameworks: the additive genetic variance-covariance matrix G (quantitative genetics), summarizing the genetic architecture of a set of traits; the distributions of phenotypic and fitness effects of new mutations (population genetics), which together with allele frequency dynamics determine which variants segregate; and the regulatory network topology that conditions both (systems biology). This review synthesizes how the evolutionary genetics of complex traits and the systems biology perspective are related: network topology constrains the distributions of phenotypic and fitness effects of new mutations, influencing the genotype-phenotype map. Then, over evolutionary time, stabilizing selection can reshape topology for increased robustness. Empirical support for these processes is reviewed: genome-scale perturbation experiments, network simulations, and evidence that stabilizing selection progressively sculpts regulatory networks toward additivity. Perspectives and future research directions are discussed.

## 1 Introduction

Complex traits have a polygenic basis: their variation maps to many loci with a broad distribution of effect sizes, and their evolution under selection typically proceeds through coordinated allele frequency shifts (Pritchard et al., 2010; Barghi et al., 2019; Yeaman, 2022). The genetic architecture underlying this polygenicity remains poorly understood despite genomic advances (Tautz et al., 2026), and can be described at three different scales. Quantitative genetics summarizes it through the genetic variance-covariance matrix G and the breeder’s equation, treating individual loci as interchangeable contributors to additive variance (Fisher, 1918; Lande, 1976; Barton et al., 2017). Population genetics models allele-frequency change under mutation, selection, and drift, deriving the distribution of fitness effects (DFE) and the relationship between fitness effect size and allele frequency from the mutation-selection balance (Eyre-Walker and Keightley, 2007). Systems biology, by studying the structure and dynamics of complex biological systems such as regulatory, metabolic, and signalling networks, offers a complementary view of the genotype-phenotype map. Applied to evolutionary genetics, this perspective asks what molecular architecture generates these distributions: gene regulatory networks, core and peripheral genes, pathway-level organization (Fagny and Austerlitz, 2021). The omnigenic model (Boyle et al., 2017) exemplifies this framing: the systems biology observations of dense regulatory connectivity is studied largely from a quantitative-genetic viewpoint, to explain observed GWAS patterns. Each tradition looks at the genotype-phenotype map at a different organizational scale, and finding a common model to unify them can be challenging. Yet the three descriptions are not independent: G and the DFE are complementary summaries of the same underlying mutation effects (i.e., G the allele-frequency-weighted view at a given time, the DFE the intrinsic fitness view) while the distribution of new mutational effects itself (sometimes summarized as the mutational covariance matrix M ) is upstream of both. Table 1 gathers the key terms from these three traditions used across this review. Network topology constrains the phenotypic and fitness effects of new mutations, shaping M and therefore both the DFE and G ; phenotypic effects define the genotype-phenotype map while fitness effects determine which variants persist. Furthermore, selection on the phenotype can reshape network topology over evolutionary time.

**TABLE 1:** Glossary of key terms. Brief definitions of important concepts used across this review.

Term	Definition
*cis/trans* regulation	*cis*-regulatory variants act locally on the same allele or transcript (e.g., promoters, enhancers); *trans*-regulatory variants act through diffusible factors on distant targets
Core/peripheral gene	In the omnigenic model, core genes act directly in trait-relevant pathways; peripheral genes contribute indirectly via *trans*-regulatory connections to core genes
Distribution of fitness effects (DFE)	Distribution of selection coefficients of new mutations, mapping mutations directly to fitness
Distribution of phenotypic effects	Distribution of trait-change magnitudes produced by new mutations, independent of fitness
Functional epistasis	Nonlinear interaction between alleles at different loci on the molecular phenotype, e.g., through regulatory architecture; a property of the genotype-phenotype map
Functional module	Densely interconnected subset of a GRN corresponding to a coherent biological function; constrains pleiotropy by limiting cross-pathway propagation
G matrix	Additive genetic variance-covariance matrix of segregating variation in a population. Governs the short-term selection response through the breeder’s equation $\Delta \bar{z}=G\beta$
Gene regulatory network (GRN)	Network of regulatory interactions among genes; edges typically represent *trans*-regulatory effects of transcription factors or other regulators on downstream gene expression
Genotype-phenotype (GP) map	Mapping from genotypes to phenotypes; its curvature reflects nonlinear regulatory interactions and shapes both M and the DFE.
Hub gene	Gene with disproportionately many regulatory connections
M matrix	Mutational variance-covariance matrix summarising magnitudes and cross-trait correlations of phenotypic effects of new mutations
Phenotypic detectability	Property of a genetic perturbation whose downstream effect on a measured phenotype (e.g., transcriptome, organismal trait) is statistically distinguishable from background variation. Depends jointly on perturbation magnitude, propagation through regulatory networks, and measurement power
Pleiotropy	Effect of a single locus on more than one trait. Pleiotropic reach is the number of traits a mutation in a given gene perturbs
Portability (of polygenic scores)	Extent to which a polygenic score trained in one population predicts accurately in another
Statistical epistasis	Observed non-additive effects of interaction between alleles at different loci on the phenotype

This review first examines what population and quantitative genetics reveal about the statistical structure of polygenic traits, and how models based on molecular networks give these distributions a biological basis. Then, it reviews recent empirical work on the mutual influence between network topology and the structure of mutation effects.

## 2 Evolutionary genetics models of complex trait evolution

Models of polygenic architecture characterize how genetic variation maps to phenotypic variation. In quantitative genetics, the additive component of this mapping is summarized by the genetic variance-covariance matrix G , which governs the short-term selection response through the breeder’s equation $\Delta \bar{z}=G\beta$ (Lande, 1976). Because G captures additive variance, it always predicts the short-term selection response; non-additive components (statistical epistasis, dominance) contribute only as they are converted into additive variance through changes in allele frequency (Cheverud and Routman, 1995). In the infinitesimal model (Table 2), G reduces to $V_{a}I$ : all loci contribute interchangeably, and the selection response depends only on total additive variance (Fisher, 1918; Barton et al., 2017). This approximation is robust and arises whenever loci are numerous and individual effects small, conditions under which the central limit theorem renders genotypic values approximately normal. G itself, however, is not static: it shifts with allele frequencies and with any change to the upstream mutational covariance M .

**TABLE 2:** Example models of the genetic architecture of complex traits. Each row summarizes a major framework, its intellectual tradition, how it considers the genetic architecture underlying complex traits, and a brief description. DFE = distribution of fitness effects; QG = quantitative genetics; PG = population genetics; SB = systems biology.

Model	Tradition	Genetic architecture	Description
Infinitesimal (Fisher, 1918; Barton et al., 2017)	QG	All loci contribute equally to additive variance	Predicts selection response from total additive variance assuming many loci of small effect
Fisher’s geometric model (Fisher, 1930; Orr, 1998)	PG/QG	DFE derived from mutation vectors in n -dimensional phenotype space under stabilizing selection	Models each mutation as a displacement in phenotype space; predicts the DFE shape and the frequency-effect relationship from evolutionary forces
Scaling laws (Simons et al., 2025)	PG	Two scaling parameters: Mutational target size L and per-site heritability $h^{2}/L$	Empirical parameterization of Fisher’s geometric model across 95 United Kingdom biobank traits. Shows trait-specific architectures are highly similar
Omnigenic (Boyle et al., 2017)	SB	Core genes (direct) + peripheral genes (*trans*-regulatory); most $V_{a}$ from periphery	Explains why focused molecular traits yield thousands of GWAS hits through dense regulatory interconnection. Binary core/periphery split
Modular/pathway-structured (Fagny and Austerlitz, 2021; García-González and O’Reilly, 2026)	SB	Risk stratified across functionally coherent pathway modules; modularity constrains pleiotropy	Refines the omnigenic model by replacing the binary split with modular internal structure
Quantitative omnigenic (QOM) (Ružičková et al., 2024)	SB/QG	*Cis + trans* effects propagating through network topology ( ≤3 steps)	Makes the omnigenic model quantitative: Derives G from network topology via *trans*-regulatory propagation

Population genetics models allele-frequency change under mutation, selection, and drift, but typically without an explicit phenotypic model: the DFE maps mutations directly to fitness effects. At mutation-selection balance, the DFE determines which alleles segregate and at what frequencies (Eyre-Walker and Keightley, 2007). Models of polygenic adaptation illustrate this phenotype-free approach: selection drives coordinated allele frequency shifts whose dynamics depend on the DFE and population size, but the trait under selection remains implicit (e.g., Höllinger et al., 2019; Höllinger 2023).

Fisher’s geometric model (FGM; Table 2) makes the link from mutations to phenotype explicit, modeling mutations as phenotypic displacements from a phenotypic optimum from which the DFE is then derived. Each new mutation is a vector of effects across n phenotypic dimensions, with selection coefficient $s\propto \sum_{i}b_{i}^{2}$ , where $b_{i}$ is the effect on trait i (Fisher, 1930; Orr, 1998). Pleiotropy constrains the overall distribution of phenotypic effects to be leptokurtic: most mutations have small net effects, with a heavy tail of larger effects. This heavy-tailed distribution is supported by recent genomic data analyses: a saturated genome-wide association study (GWAS) for human height identifies 12,111 independent SNPs exhibiting exactly this structured, heavy-tailed distribution (O’Connor, 2021; Yengo et al., 2022), strongly pathway-structured rather than diffuse (Mostafavi, 2025). Note that these analyses characterize phenotypic effect sizes (heritability explained), which does not necessarily match the DFE when the fitness function is nonlinear, as under stabilizing selection. Simons et al. (2025) show that this pattern generalizes (Table 2): the architecture of 95 highly polygenic United Kingdom Biobank traits are highly similar and map to two scaling parameters related to Fisher’s geometric model, the mutational target size L and the per-site heritability $h^{2}/L$ .

These models specify what the distribution of mutation effects looks like and how it scales across traits. They do not, however, fully explain why the distribution takes the shape it does. In particular, population and quantitative genetics models do not explain why many traits appear mostly additive despite being generated by nonlinear regulatory networks. Part of the answer lies within quantitative genetics itself: functional epistasis does not necessarily translate into statistical epistasis, and non-additive variance components are systematically converted into additive variance as allele frequencies move away from intermediate values (Cheverud and Routman, 1995). The very structure of variance decomposition biases the apparent contribution toward additivity, so additive variance does not imply additive gene action (Huang and Mackay, 2016). A systems biology perspective complements this by asking what network architectures generate the underlying functional nonlinearities in the first place.

## 3 Regulatory network structure as mechanistic models of genetic architecture

The “omnigenic” model (Table 2; Boyle et al., 2017) is a significant step to provide the polygenic effect-size distribution an underlying biological mechanism. It distinguishes “core” genes (those in trait-relevant pathways) from “peripheral” genes that contribute indirectly through gene regulatory networks (GRNs). Because GRNs are densely interconnected, peripheral genes outnumber core genes, therefore most heritability comes from the aggregate small effects of peripheral genes (Boyle et al., 2017; Liu et al., 2019; O’Connor et al., 2019). The scale-free topology of GRNs, i.e., the observation that a few “hub” genes have many connections while most genes have few, provides a topological basis for this core-peripheral asymmetry. Indeed, hubs are expected to be under strong constraint, while the peripheral majority remains accessible to selection (Fagny and Austerlitz, 2021). Empirical perturbation data support this core-peripheral distinction: Ratajczak et al. (2025) show that core genes respond coordinately to perturbations and exhibit frequent functional epistatic interactions, suggesting that non-additive effects concentrate among network-central genes. Functional network modularity (Table 2) further limits cross-pathway effects, replacing the extreme global pleiotropy implied by an unstructured omnigenic model with pleiotropic effects restricted to specific pathway modules (Fagny and Austerlitz, 2021; García-González and O’Reilly, 2026). It should be noted that functional modules (defined by shared biological function) and variational modules (defined by covarying mutational effects) do not necessarily coincide, e.g., in *Drosophila serrata* where Gene Ontology functional groupings predicted variational modularity in only ∼ 38% of cases (Collet et al., 2018). This suggests that pleiotropic constraints can differ across genes within the same functional pathway.

The modular refinement reconciles two observations that initially appear in tension: GWAS hits are pervasive and near-uniform across the genome (Visscher et al., 2017; Watanabe et al., 2019), yet complex-trait risk often clusters in functionally coherent pathways. Indeed, empirically, complex traits vary in their degree of modularity: type-2 diabetes risk loci cluster in biologically coherent pathway modules (McCarthy, 2017), while schizophrenia GWAS signals accumulate in genes most connected to risk-enriched co-expression clusters (Borcuk et al., 2024). Because modules limit the pleiotropic reach of mutations, the trait-specific mutational target concentrates within functionally relevant modules, even though contributions from *trans*-regulatory pathways extend broadly across the genome: pervasiveness then reflects the density of *trans*-regulatory connectivity, while modularity concentrates the impact of any one variant within trait-relevant pathways. In this context, it is important to note that GWAS preferentially picks up large-effect variants that often correspond to core genes, while peripheral-gene contributions accumulate across many *trans*-regulatory steps and remain difficult to resolve as individual loci.

A shared limitation of the omnigenic and modular models is that they are mainly verbal. The quantitative omnigenic model (QOM; Table 2) of Ružičková et al. (2024) is an attempt to model the phenotype as the sum of direct *cis* effects and *trans* effects that propagate through successive regulatory steps, with each step governed by the network’s observed topology. Applied to yeast gene expression, the QOM explains 50% of heritable variance with up to three propagation steps and achieves predictions consistent with traditional GWAS with orders of magnitude fewer parameters, a significant step in mapping network topology to the structure of the G matrix using real data. More generally, this maps the core/peripheral split onto the variance-covariance matrices: peripheral genes populate G with many small additive contributions, while core genes inject low-rank correlation structure into the upstream M . G then derives from M as selection, drift, and migration shape the allele frequencies of the segregating variants. The translation is useful because it lets G be predicted from network data without exhaustive phenotyping, explains cross-population G variation (Steppan et al., 2002; Arnold et al., 2008) and the limited portability of polygenic scores (e.g., Mostafavi et al., 2020), and identifies which features best capture the influence of biological networks topology on complex trait evolution.

## 4 The interaction between network topology and intrinsic mutation effects

The models reviewed above describe which classes of genes contribute to trait variation, but treat network topology as fixed. In practice, topology both shapes mutation effects and is itself shaped by selection.

To capture how topology shapes mutation effects, the quantitative genetics tradition distinguishes M , the mutational variance-covariance matrix for new mutations (Lande, 1980; Jones et al., 2007), from G , its allele-frequency-weighted counterpart for segregating variation. Regulatory curvature in the genotype-phenotype map shapes M directly: core genes (with broad downstream reach) generate phenotypic effects correlated across the traits their targets influence, aligning M ’s principal axes along a few pleiotropic dimensions (Cheverud, 1984; Hansen and Wagner, 2001), while peripheral genes contribute more independent, lower-dimensional perturbations. M thus acts as a developmental constraint on the variation introduced each generation; G inherits this structure but is further reshaped by evolutionary forces. The empirical results that follow can therefore be read as observations on M .

**Network topology conditions mutation effects**. A mutation’s network position partly determines how it translates into a phenotypic effect through the genotype-phenotype map, and the phenotypic effect in turn conditions the fitness effect. Pouzet and Le Rouzic (2025) simulated evolution across different gene regulatory network topologies and found that topology has more influence on the DFE than mutation type (coding versus regulatory). In scale-free networks, coding mutations are more pleiotropic and overrepresented in both tails of the DFE, while in other -less common-topologies, this pattern reverses. These specific results were obtained under an evolutionary scenario starting from a ∼ 50% drop in fitness; under more typical adaptation from high-fitness genotypes, the beneficial tail of the DFE is expected to remain approximately exponential regardless of topology (Gillespie, 1984; Orr, 2003), so the topology effect documented here applies primarily to the bulk distribution rather than the beneficial right tail. Stone et al. (2025) showed that heritability clusters in tissue-specific regulatory hubs, which simultaneously amplify focal-trait phenotypic effects and buffer pleiotropic consequences. This hub-periphery asymmetry appears to be taxonomically pervasive and conditions the evolutionary paths available to complex traits. High-connectivity genes tend to be under stronger negative selection due to their pleiotropic reach (Tyler et al., 2009; Luisi et al., 2012; Fagny and Austerlitz, 2021), while positive selection preferentially targets the lower-pleiotropy periphery, where mutations can alter specific traits with fewer collateral effects (Luisi et al., 2015; Gouy et al., 2017). To understand the mechanistic basis of these observations and their generality, studies should focus on how perturbations propagate through the network. Ota et al. (2026) combined Perturb-seq data with loss-of-function burden tests to trace how perturbations at peripheral genes propagate through coregulated gene sets, directly measuring the *trans*-regulatory component of the genotype-phenotype map that the omnigenic model postulated. Aguirre et al. (2025) couple the empirical observation that only ∼ 41% of CRISPRi perturbations have a measurable effect on the expression of other genes (based on the genome-scale Perturb-seq data from Replogle et al., 2022) with simulations across synthetic networks, showing that sparsity, modularity, and hub structure limit propagation and consequently how mutations contribute to the phenotype. This identifies network position rather than perturbation efficacy as the main determinant of phenotypic detectability.

**Natural selection shapes network topology**. This link operates on a longer timescale but has growing simulation support. Ciliberti et al. (2007) showed that robustness in GRNs is an evolvable trait: the space of network architectures can be traversed through gradual changes in regulatory interactions. More recently, Petit et al. (2023) simulated GRN evolution under correlated stabilizing selection and found that selection can reshape network topology by shortening the distance between genes in the regulatory network. However, the relationship between network-level topology and gene expression dynamics is subject to a genuine scale-mismatch concern: expression dynamics are intrinsically a gene-level property, while whole-network statistics summarise connectivity at a much coarser resolution. Motif-level topology, an intermediate scale, would therefore seem the more natural comparator. Testing this directly, Petit et al. (2026) show that gene expression plasticity correlates with regulatory complexity (number of regulators per gene) but not with specific network motifs, suggesting that the topological features through which selection acts operate at a scale coarser than individual motifs but finer than whole-network connectivity statistics, the number of regulators converging on a gene being one such intermediate-scale feature.

## 5 Discussion

A central idea emerging from the work reviewed here is that the phenotypic and fitness consequences of a mutation are not solely determined by the gene it affects, but are conditioned by its position within a biological network. This framing parallels Lewontin’s longstanding argument that gene, organism, and environment are mutually constitutive (Lewontin, 2000): a gene’s regulatory neighbourhood functions as part of its “developmental” environment, both being mutually constitutive. Topology shapes how perturbations propagate, which traits are affected, and how strongly. A concrete implication is that the same mutation can have different phenotypic and fitness effects depending on the network context, whether across cell types, tissues, or populations with divergent regulatory architectures. The non-portability of polygenic scores across populations (Mostafavi, 2025) and the population-specific nature of epistatic effects (Bank, 2022) are consistent with this view.

Several methodological developments can be considered in the future to better understand the relationship between network topology and phenotypic variation. As discussed above, the quantitative omnigenic model (Ruzickova et al., 2024) derives the mutational and additive variance-covariance structure directly from network topology: each gene’s effect on a focal phenotype is modelled as the sum of its *cis* and *trans* contributions propagated through observed regulatory edges, yielding predictions for individual mutation effects that aggregate into M and G . This regulatory-architecture-aware approach could be extended to organismal models. Methodologically, connecting network topology to population-level variation will require the development of analytical approaches that integrate regulatory network structure with allele frequency data and phenotypic measurements. Furthermore, cell-type-resolved perturbation atlases (e.g., Dixit et al., 2016; Replogle et al., 2022) are beginning to provide the regulatory network maps needed at the cell-type and tissue scale, at which gene regulation actually operates. This resolution matters because regulatory architecture differs substantially across cell types: a mutation that propagates through a hub in one tissue may have no propagation route in another, so the network-conditioned mutation effects described throughout this review should differ across tissues. These atlases therefore supply the cell-type-specific structural input that any tissue-aware prediction of M and G requires, and make tissue-specific mutation-effect predictions empirically testable. Finally, large-scale experiments, including parallel selection experiments or GWAS in environmental perturbation experiments will help uncover how the polygenic architecture of complex traits conditions their variability and evolution (Tautz et al., 2026). More fundamentally, this review has treated network topology as a relatively stable property, but how evolvable, generalizable across species, and plastic in response to environmental change network structures actually are remains poorly understood. Addressing these questions is essential to determine if the topology-mediated effects described here are robust features of genetic architecture or if they depend on specific regulatory contexts. This question connects directly to the G / M framework of the quantitative genetics tradition: G and M are known to vary substantially across populations and species (Steppan et al., 2002; Arnold et al., 2008), and if network topology conditions M as argued above, then variation in regulatory architecture should leave a signature in cross-population G / M comparisons. Developmental models in which network structure shapes the production of heritable variation make this expectation explicit (Draghi and Wagner, 2008), and developmental systems drift (True and Haag, 2001) provides an extreme case where closely related lineages can converge on the same phenotype through divergent regulatory wiring, with consequences for hybrid incompatibilities and speciation.

In sum, understanding the genetic architecture of complex traits requires moving beyond gene-centric models toward approaches that capture how network topology mediates the mapping from genotype to phenotype. The convergence of advances in sequencing methods, diverse population-scale GWAS in various organisms, and network-aware analytical methods should lead to a more comprehensive understanding of the evolution of complex traits.
